# High-level resistance to non-nucleos(t)ide reverse transcriptase inhibitor based first-line antiretroviral therapy in Ghana; A 2017 study

**DOI:** 10.3389/fmicb.2022.973771

**Published:** 2022-08-25

**Authors:** Prince Kofi Parbie, Christopher Zaab-Yen Abana, Dennis Kushitor, Theodore Worlanyo Asigbee, Nana Afia Asante Ntim, Gifty Addo-Tetebo, Maclean Richard Darko Ansong, Sampson Badu Ofori, Taketoshi Mizutani, Lucky Ronald Runtuwene, Masako Nishizawa, Koichi Ishikawa, Hiroshi Kiyono, William Kwabena Ampofo, Tetsuro Matano, Evelyn Yayra Bonney, Tadashi Kikuchi

**Affiliations:** ^1^AIDS Research Center, National Institute of Infectious Diseases, Tokyo, Japan; ^2^Noguchi Memorial Institute for Medical Research, University of Ghana, Accra, Ghana; ^3^West African Center for Cell Biology of Infectious Pathogens (WACCBIP), University of Ghana, Accra, Ghana; ^4^Department of Biochemistry, Cell and Molecular Biology, College of Basic and Applied Sciences, University of Ghana, Accra, Ghana; ^5^Joint Research Center for Human Retrovirus Infection, Kumamoto University, Kumamoto, Japan; ^6^Eastern Regional Hospital Koforidua, Ghana Health Service, Koforidua, Ghana; ^7^The Institute of Medical Science, The University of Tokyo, Tokyo, Japan; ^8^Future Medicine Education and Research Organization, Institute for Global Prominent Research, Graduate School of Medicine, Chiba University, Chiba, Japan; ^9^Department of Medicine, Chiba University-University of California San Diego Center for Mucosal Immunology, Allergy and Vaccines (cMAV) University of California San Diego, San Diego, CA, United States

**Keywords:** HIV-1, drug resistance, Ghana, nucleos(t)ide reverse transcriptase inhibitors, non-nucleos(t)ide reverse transcriptase inhibitors, protease inhibitors, integrase strand transfer inhibitors

## Abstract

Expanding access to effective antiretroviral therapy (ART) is a major tool for management of Human Immunodeficiency Virus (HIV) infection. However, rising levels of HIV drug-resistance have significantly hampered the anticipated success of ART in persons living with HIV (PLWH), particularly those from Africa. Though great strides have been made in Ghana toward achieving the UNAIDS “95-95-95” target, a substantial number of PLWH receiving ART have not attained viral suppression. This study investigated patterns of drug resistance mutations in ART naïve as well as ART-experienced PLWH receiving first-line regimen drugs from Ghana. In a cross-sectional study, blood samples were collected from HIV-1 infected adults (≥18 years) attending HIV/AIDS clinic at the Eastern Regional Hospital, Koforidua, Ghana from September to October 2017. Viral RNA isolated from plasma were subjected to genotypic drug resistance testing for Protease Inhibitors (PI), Reverse Transcriptase Inhibitors (RTI), and Integrase Strand Transfer Inhibitors (INSTI). A total of 95 (84 ART experienced, 11 ART naïve) HIV-1 infected participants were sampled in this study. Sixty percent (50/84) of the ART-experienced participants were controlling viremia (viral load < 1,000 copies/ml). Of the 95 patient samples, 32, 34, and 33 were successfully sequenced for protease, reverse-transcriptase, and integrase regions, respectively. The dominant HIV-1 subtypes detected were CRF02_AG (70%), and A3 (10%). Major drug resistance associated mutations were only detected for reverse transcriptase inhibitors. The predominant drug resistance mutations were against nucleos(t)ide reverse transcriptase inhibitors (NRTI)—M184V/I and non-nucleos(t)ide reverse transcriptase inhibitors (NNRTI)—K103N. In the ART-experienced group, M184V/I and K103N were detected in 54% (15/28) and 46% (13/28) of individuals, respectively. Both mutations were each detected in 33% (2/6) of ART naïve individuals. Multiclass resistance to NRTI and NNRTI was detected in 57% of ART-experienced individuals and two ART naïve individuals. This study reports high-level resistance to NNRTI-based antiretroviral therapy in PLWH in Ghana. However, the absence of major PI and INSTI associated-mutations is a good signal that the current WHO recommendation of Dolutegravir in combination with an NRTI backbone will yield maximum benefits as first-line regimen for PLWH in Ghana.

## Introduction

Infection with HIV-1 continues to be a major global public health issue. Globally, over two thirds of the estimated 37.7 million people living with HIV (PLWH) as of 2020 were from Sub-Saharan Africa (World Health Organization, 2021b). Indeed, increasing access to effective ART is one of the key tools for HIV/AIDS prevention and control. Thus, the Joint United Nations Program on HIV/AIDS (UNAIDS) proposed a global target of diagnosing 95% of all HIV-positive individuals, providing ART for 95% of people who know their status, and achieving viral suppression in 95% of those receiving treatment by 2030, termed 95-95-95 (UNAIDS, 2014). In a concerted effort to meet this global target, Ghana, in 2016, adopted a national strategy of treating all HIV infected individuals, regardless of clinical status or CD4 cell count, with a triple drug regimen [Ghana Health Service (GHS), National AIDS/STI Control Programme, 2016]. The anticipated impact of increased access to ART has been hampered by increasing levels of HIV drug-resistance in low- or middle income countries (LMIC) such as found in Sub-Saharan Africa (Aghokeng et al., 2011; Gupta et al., 2018; World Health Organization, 2019). This is attributable to several factors, including poor adherence, inadequate virologic monitoring, and lack of drug resistance testing.

In Ghana, the initiation of treat all policy has significantly expanded access to ART, however, only 66% of PLWH receiving ART have achieved viral suppression (UNAIDS, 2019). Though pre-treatment/transmitted drug resistance has consistently been reported to be low in Ghana, acquired drug resistance remains a major concern (Nii-Trebi et al., 2013; Martin-Odoom et al., 2018; Deletsu et al., 2020; Obeng et al., 2020), due to lack of routine viral load testing and drug resistance testing to guide the selection of drugs. At the time of sampling, first-line regimens in Ghana consisted in NRTI and NNRTIs only, while the preferred second- and third-line drug regimen in Ghana included PI and INSTI, respectively [Ghana Health Service (GHS), National AIDS/STI Control Programme, 2016]. With the current World Health Organization recommended Dolutegravir-based first-line regimen in combination with an NRTI backbone (World Health Organization, 2021a), it is critical to investigate resistance profiles of patients in order to provide country-specific data to guide optimum drug choices. This study reports on patterns of resistance observed against protease (PR), reverse transcriptase (RT), and integrase (IN) inhibitors in both ART-naïve and -experienced PLWH in Ghana.

## Materials and methods

### Study design

In this cross-sectional study, we enrolled HIV-1 infected adults (≥18 years) attending an HIV/AIDS clinic at the Eastern Regional Hospital, Koforidua, Ghana. All participants resided in communities located within an area of 1,200 km^2^ in the Eastern region of Ghana. Blood samples were collected from consenting participants between September and October 2017. Clinical and demographic data were obtained from hospital records for all consenting participants.

### HIV-1 drug-resistance genotyping

Viral RNA was isolated from 200 μl of plasma samples using High Pure Viral RNA Kit (Roche, Mannheim, Germany) following the manufacturer’s protocol, and stored at −80°C until use. One-step reverse transcription PCR (RT-PCR) was performed for the PR, RT, and IN regions of the polymerase gene, using PrimeScript™ II High Fidelity One Step RT-PCR Kit (Takara, Japan) following the manufacturer’s protocol. This was followed by nested PCR using KOD–Plus–Ver.2 (Toyobo, Japan) following manufacturer’s recommendation. Samples were then purified with the FavorPrep GEL/PCR Purification Kit (Favorgen, Taiwan), and sequenced using Big Dye Terminator cycle sequencing kit version 3.1 (Applied Biosystems Inc., United States) and 3730/3730xl DNA Analyzer (Applied Biosystems Inc., United States). Details of primers (Kotaki et al., 2014; ANRS, 2015; Ode et al., 2015) used are described in Supplementary Table 1. The RECall (version 2.30) HIV-1 sequencing analysis tool (Woods et al., 2012) was used for sequence editing and assembly. Subtyping was performed using REGA (version3.46; Pineda-Peña et al., 2013) and Recombinant Identification Program (RIP, version 3.0; Siepel et al., 1995) based on PR and RT sequences. All mutations listed as major mutation, minor mutation, or mutation with a penalty score in any of the following three databases were analyzed: the Stanford University HIV drug resistance database version 9.0, The Agence Nationale de Recherches sur le SIDA (French National Agency for AIDS Research) drug resistance mutations list, and The International AIDS Society, United States drug resistance mutations list (Liu and Shafer, 2006; Wensing et al., 2019; ANRS, 2021). In this study, we classified major mutations as those defined in the 2019 edition of the International Antiviral Society–USA (IAS–USA) drug resistance mutations list (Wensing et al., 2019). Mutations for drug resistance surveillance are classified as defined by WHO list of mutations for surveillance of transmitted drug resistant HIV strains (Bennett et al., 2009; Tzou et al., 2020). Interpretation of drug resistance mutations was done using the Stanford University HIV drug resistance database version 9.0 (Liu and Shafer, 2006).

### Statistical analysis

Statistical analyses were performed using GraphPad Prism version 9. Comparison between categorical variables between groups was performed with Fisher’s exact test. Continuous variables were assessed using Wilcoxon rank sum test. Values of *p* less than 0.05 were considered significant.

## Results

### Clinical and demographic information

A total of 95 HIV-1 infected participants were sampled in this study; majority of whom were females (72/95, 76%). Eleven (11/95) were ART naïve while 84 were receiving ART (Supplementary Figure 1). Three of the ART naïve participants had undetectable plasma viral RNA (<20 copies/ml), while seven of them had plasma viral RNA > 1,000 copies/ml with a median CD4 cell count of 265 cells/ml (IQR, 167–415). Eighty-four of the participants had been on ART for a median duration of 54 months (IQR, 20–101) with 50 (60%) of them controlling viremia (<1,000 copies/ml). The median CD4 cell count in those receiving ART but failing treatment (*n* = 34, VL ≥ 1,000 copies/ml) was 204 cells/ml (IQR, 113–464). All participants receiving ART were on first-line regimen consisting of two NRTI (Zidovudine + Lamivudine or Tenofovir disoproxil fumarate + Lamivudine) and one NNRTI (Efavirenz or Nevirapine). Majority (47/84, 56%) of participants receiving ART were on Tenofovir disoproxil fumarate + Lamivudine + Efavirenz combination first-line regimen (Table 1).

**Table 1:** Demographic and clinical characteristics of study participants.

Description	ART experienced (*n* = 84)	ART Naïve (*n* = 11)	Value of *p*
Gender, Female (%)	66(79%)	6(55%)	0.1
Age(years): median (IQR)	47 (38–52)	39 (36–48)	0.2
CD4 cell count (cells/ml): median (IQR)	389 (217–591)	396 (250–483)	0.8
Viral load (copies/ml): median (IQR)	3.5 × 10^2^ (<20–1.1 × 10^4^)	8.9 × 10^3^ (1.1 × 10^2^–3.0 × 10^4^)	0.2
Viral load <1,000 copies/ml, *n* (%)	50(60%)	4(36%)	0.2
ART duration (months): median (IQR)	54 (20–101)	ND	ND
ART regimen:			
AZT + 3TC + EFV	21(25%)		
AZT + 3TC + NVP	10(12%)		
TDF + 3TC + EFV	47(56%)		
TDF + 3TC + NVP	6(7%)		

Statistical comparison between ART groups was performed by Wilcoxon rank sum test or Fisher’s exact test. AZT, zidovudine; 3TC, lamivudine; TDF, Tenofovir disoproxil fumarate; EFV, efavirenz; NVP, nevirapine; and IQR, interquartile range.

### HIV-1 subtypes

**Figure 1:**
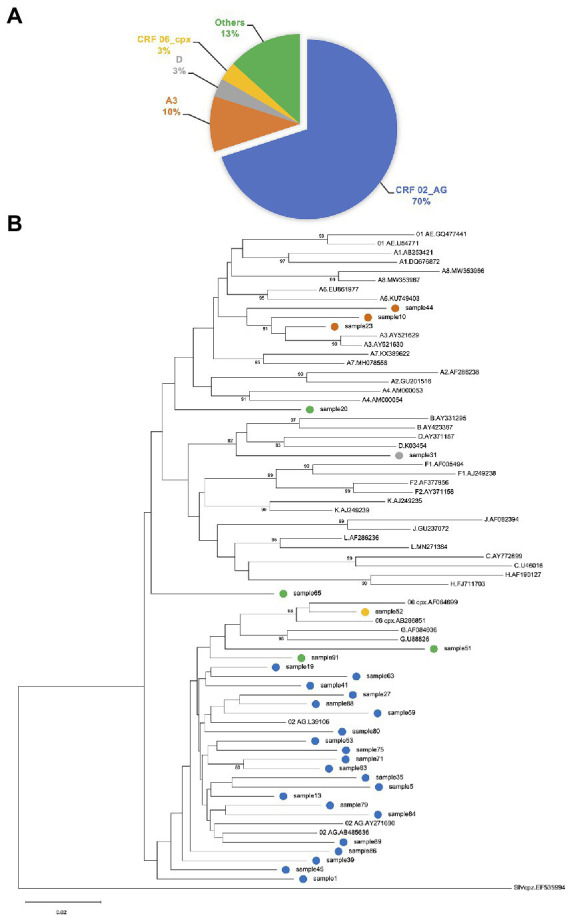
**(A)** Proportion of HIV-1 genotypes determined by protease (PR) and reverse transcriptase (RT) sequences. Subtyping was performed using REGAv3.46 and Recombinant Identification Program (RIP.v.3.0). The predominant subtype in this study was CRF02_AG (70%). Recombinants of A3 and G; A3 and CRF02_AG; A1 and G, as well as G and CRF02_AG are grouped as “Others.” **(B)** Phylogenetic inference of HIV-1 subtypes. Phylogenetic analysis of PR-RT sequences using neighbor-joining tree with the Molecular Evolutionary Genetic Analysis tool version 11 (MEGA11). Clustering pattern of study samples corroborates classification of samples by subtyping tools. Study samples are tagged with colored circles, indicating the various subtypes. References were obtained from the Los Alamos database (https://www.hiv.lanl.gov/components/sequence/HIV/). Labelling format of reference sequences = Subtype.Accession-number.

The PR, RT, and IN regions were successfully sequenced from 32, 34, and 33 of samples, respectively. Sequence success rate was higher in samples with viral loads greater than 1,000 copies/ml (*n* = 41): 28(68%), 31(76%), and 31(76%) for PR, RT, and IN, respectively. In 30 samples with both PR and RT sequences, 21(70%) were classified as CRF02_AG and 3(10%) as A3 (Figures 1A,B). CRF06_cpx and D were detected in one sample each. Likewise, recombinants of A3 and G; A3 and CRF02_AG; A1 and G, as well as G and CRF02_AG were detected in one sample each.

### Drug resistance-associated mutations

In this study, we classified major mutations as those defined in the 2019 edition of the IAS–USA drug resistance mutations list (Wensing et al., 2019). Resistance associated mutations detected in analyzed sequences are summarized in Table 2. There were no major drug resistance (DR) mutations detected for PI or INSTI. Among the 28 ART-experienced RT sequences, 16 (57%) possessed one or more major NRTI resistance mutation. All 16 ART-experienced participants possessing one or more major NRTI resistance mutation were failing treatment (VL > 1,000 copies/ml). The predominant mutation to NRTI was M184V/I; detected in 15 (54%) of ART-experienced RT sequences. Other mutations to NRTI included T215F/Y, A62V, K70E/R, K219E/Q, M41L, K65R, L74I, and D67N. One or more major NNRTI resistance mutation was detected in 75% (21/28) of ART-experienced RT sequences. K103N was the predominant (13/28, 46%) mutation to NNRTI. Other NNRTI mutations detected included G190A/S, P225H, Y181C, V106A, K101E, V108I, H221Y, E138A, Y188C/L, and M230L. No statistical difference was detected when association of major DR mutations with the different ART regimen and duration on ART were computed.

**Table 2:** Patterns of mutations detected in protease, reverse-transcriptase, and integrase regions of the HIV-1 polymerase gene.

Description	ART experienced	ART Naïve	Total
**Major NRTI resistance mutations** ^**1**^			
M184I/V	15 (54%)	2 (33%)	17 (50%)
T215F/Y	5 (18%)	0 (0%)	5 (15%)
A62V	4 (14%)	0 (0%)	4 (12%)
K70E/R	3 (11%)	0 (0%)	3 (9%)
K219E/Q	3 (11%)	0 (0%)	3 (9%)
M41L	2 (7%)	0 (0%)	2 (6%)
K65R	2 (7%)	0 (0%)	2 (6%)
L74I	2 (7%)	0 (0%)	2 (6%)
D67N	1 (4%)	0 (0%)	1 (3%)
Any major NRTI resistance mutations	16 (57%)	2 (33%)	18 (53%)
Any NRTI mutations for Drug Resistance Surveillance^2^	16 (57%)	2 (33%)	18 (53%)
**Other NRTI resistance-associated mutations**			
E44D	3 (11%)	0 (0%)	3 (9%)
T69N/S	2 (7%)	0 (0%)	2 (6%)
K70G/N	3 (11%)	0 (0%)	3 (9%)
**Major NNRTI resistance mutations**			
K103N	13 (46%)	2 (33%)	15 (44%)
G190A/S	9 (32%)	1 (17%)	10 (29%)
P225H	7 (25%)	0 (0%)	7 (21%)
Y181C	5 (18%)	1 (17%)	6 (18%)
V106A	5 (18%)	0 (0%)	5 (15%)
K101E	4 (14%)	0 (0%)	4 (12%)
V108I	3 (11%)	1 (17%)	4 (12%)
H221Y	3 (11%)	0 (0%)	3 (9%)
E138A	2 (7%)	1 (17%)	3 (9%)
Y188C/L	2 (7%)	0 (0%)	2 (6%)
M230L	1 (4%)	0 (0%)	1 (3%)
Any major NNRTI resistance mutations	21 (75%)	2 (33%)	23 (68%)
Any NNRTI mutations for Drug Resistance Surveillance	21 (75%)	2 (33%)	23 (68%)
**Other NNRTI resistance-associated mutations**			
V179E/I	12 (43%)	1 (17%)	13 (38%)
V90I	5 (18%)	1 (17%)	6 (18%)
A98G/S	4 (14%)	1 (17%)	5 (15%)
F227L	4 (14%)	0 (0%)	4 (12%)
V106I/T	2 (7%)	1 (17%)	3 (9%)
L234I	1 (4%)	0 (0%)	1 (3%)
K238T	1 (4%)	0 (0%)	1 (3%)
K101R	0 (0%)	1 (17%)	1 (3%)
	(***n*** = 27)	(***n*** = 5)	(***n*** = 32)
**Major PI resistance mutations**			
Any major PI resistance mutations	0 (0%)	0 (0%)	0 (0%)
Any PI mutations for Drug Resistance Surveillance	0 (0%)	0 (0%)	0 (0%)
**Other PI resistance-associated mutations**			
M36I/V	27 (100%)	5 (100%)	32 (100%)
H69K	26 (96%)	5 (100%)	31 (97%)
L89I/M	26 (96%)	5 (100%)	31 (97%)
K20I/R	25 (93%)	5 (100%)	30 (94%)
G16E	10 (37%)	2 (40%)	12 (38%)
L10I/V	9 (33%)	1 (20%)	10 (31%)
L63P	8 (30%)	2 (40%)	10 (31%)
I64L/M	4 (15%)	1 (20%)	5 (16%)
V11I	4 (15%)	0 (0%)	4 (13%)
A71T	1 (4%)	0 (0%)	1 (3%)
V82I	1 (4%)	0 (0%)	1 (3%)
V77I	0 (0%)	1 (20%)	1 (3%)
	(***n*** = 27)	(***n*** = 6)	(***n*** = 33)
**Major INSTI resistance mutations**			
Any major INSTI resistance mutations	0 (0%)	0 (0%)	0 (0%)
Any INSTI mutations for Drug Resistance Surveillance	0 (0%)	0 (0%)	0 (0%)
**Other INSTI resistance-associated mutations**			
L74I/M	6 (22%)	1 (17%)	7 (21%)
E157Q	3 (11%)	0 (0%)	3 (9%)
T97A	2 (7%)	0 (0%)	2 (6%)
D232N	1 (4%)	0 (0%)	1 (3%)

1 Major mutations are classified as defined by the 2019 edition of the IAS-USA drug resistance mutations list.

2 Mutations for drug resistance surveillance are classified as defined by WHO list of mutations for surveillance of transmitted drug resistant HIV strains.

Among the six ART naïve patient RT sequences, major DR mutations were detected in two patients. Both patients possessed M184V/I, the only major NRTI resistance mutation detected in ART naïve sequences. For major NNRTI mutations, one patient possessed only K103N while the other possessed K103N in addition to V108I, Y181C, G190A, and E138A mutations.

**Figure 2:**
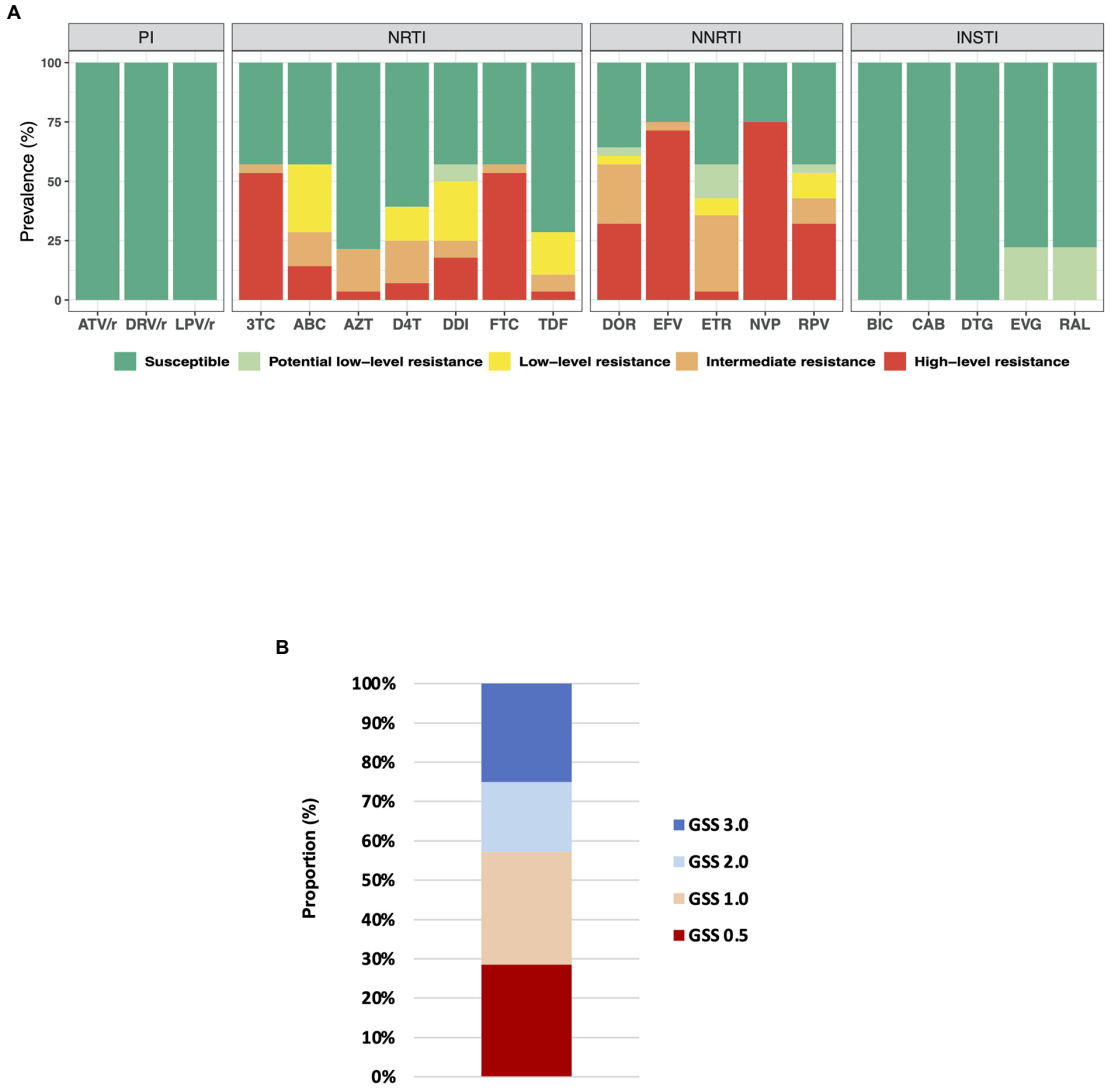
**(A)** Drug resistance profile in ART-experienced individuals. Drug resistance mutations were interpreted using Stanford University HIV drug resistance database version 9.0. ATV/r, ritonavir-boosted Atazanavir; DRV/r, ritonavir-boosted Darunavir; LPV/r, ritonavir-boosted Lopinavir; 3TC, lamivudine; ABC, Abacavir; AZT, zidovudine; D4T, Stavudine; DDI, Didanosine; FTC, Emtricitabine; TDF, Tenofovir disoproxil fumarate; DOR, Doravirine; EFV, Efavirenz; ETR, Etravirine; NVP, nevirapine; RPV, Rilpivirine; BIC, Bictegravir; CAB, Cabotegravir; DTG, Dolutegravir; EVG, Elvitegravir; and RAL, Raltegravir. **(B)** Genotypic susceptibility score (GSS) for ART-experienced participants; *n* = 28. The GSS was obtained using the Stanford University HIV genotypic resistance interpretation system. We classified patients as follows; No resistance or potential low-level resistance (Stanford penalty score < =14): GSS = 1; Intermediate resistance or low-level resistance (Stanford penalty score 15–59): GSS = 0.5; High-level resistance (Stanford penalty score > =60): GSS = 0.

A profile of inferred levels of resistance to commonly used antiretrovirals is presented in Figure 2A. Seventy-five percent (21/28) of ART-experienced individuals showed high-level resistance to at least one antiretroviral drug. We detected multiclass resistance in 16 (57%) of ART-experienced individuals. For NRTI, high−level resistance to Lamivudine, Emtricitabine, Didanosine, Abacavir, Stavudine, Abacavir, and Tenofovir disoproxil fumarate was detected in 15 (54%), 15(54%), 5(18%), 4(14%), 2(7%), 1(4%), and 1(4%) patients, respectively. Also, for NNRTI, high-level resistance to Nevirapine, Efavirenz, Doravirine, Rilpivirine, and Etravirine was detected in 21(75%), 20(71%), 9(32%), 9(32%) and 1(4%) patients, respectively. Multiclass resistance was also observed in the two ART naïve patients with one of them showing high-level resistance to all NNRTIs.

## Discussion

Increasing rates of HIV drug-resistance has impeded the progress of ART in PLWH, particularly those from Africa. As of 2018, viral suppression was achieved in 66% of PLWH receiving ART in Ghana (UNAIDS, 2019). Likewise, in our study, we recruited participants between September and October 2017 and observed that 60% (50/84) of participants receiving ART had achieved viral suppression. This was still below the expected target of 90% by 2020 (UNAIDS, 2014) and leaves much to be desired. Interestingly, the virologic failure cases had been receiving first-line regimen ART for average period of 65 months (standard deviation ±43). These cases would have been detected and given the needed attention if viral load testing was routinely performed as recommended in the national ART guidelines [Ghana Health Service (GHS), National AIDS/STI Control Programme, 2016]. However, logistic constraints such as frequent breakdown of viral load equipment and sporadic shortage of reagents have hindered the full implementation of the guidelines nationwide (UNAIDS, 2019) and resulted in deficiencies in the effectiveness of ART program as observed in our study.

In Western and Central Africa, CRF02_AG has been the predominant HIV-1 subtype (Hemelaar et al., 2019). Consistent with previous studies (Delgado et al., 2008; Nii-Trebi et al., 2013, 2017; Martin-Odoom et al., 2018; Villa et al., 2018; Deletsu et al., 2020; Obeng et al., 2020), we report CRF02_AG as the predominant (70%) HIV-1 subtype in this study population from Ghana.

We report a high frequency of major mutations associated with resistance to NRTI (57%) and NNRTI (75%) in PLWH receiving ART. The pattern and frequency of the most common mutations, such as M184V/I, K103N, T215F/Y, G190A/S, P225H, Y181C, and V106A were consistent with reports from Ghana and other sub-Saharan African countries (Hamers et al., 2012; Nii-Trebi et al., 2013; Martin-Odoom et al., 2018; Villa et al., 2018; World Health Organization, 2019; Deletsu et al., 2020). The non-polymorphic mutation K103N is selected in patients receiving Nevirapine or Efavirenz and reduces susceptibility to these drugs (Melikian et al., 2014). Likewise, M184V/I is selected by Lamivudine/Emtricitabine and reduces susceptibility to these drugs (Whitcomb et al., 2003; Melikian et al., 2012; Quercia et al., 2018). The high occurrence of M184V/I and K103N in our patients is not surprising since all ART-experienced participants were receiving regimens containing Lamivudine and Efavirenz or Nevirapine (Table 1). As expected, the high prevalence of M184V/I resulted in occurrences of high resistance to Lamivudine and Emtricitabine, while most of the patients were susceptible to Zidovudine and Tenofovir disoproxil fumarate (Figure 2A). This is a well-known phenomenon in virus isolates harboring M184V/I mutation (Quercia et al., 2018). Thymidine analogue mutations (TAMs) including M41L, D67N, K70R, L210W, T215Y/F, and K219Q/E are known to confer reduced susceptibility to most approved NRTI (Whitcomb et al., 2003). In this study, the rate of TAMs was 29% (8/28). The most common TAM detected was T215F/Y, detected in 18% (5/28) patients. Other TAMs detected include M41L, D67N, K70R, and K219Q/E. Consistent with a previous study reporting higher prevalence of TAMs in individuals on long-term ART (>36 months) from Ghana (Nii-Trebi et al., 2013), all the TAMs detected in this study were in individuals receiving ART for a minimum of 40 months. TAMs are reported to be common in individuals failing tenofovir disoproxil fumarate-based regimens in sub-Saharan Africa (Gregson et al., 2017; Henerico et al., 2022). Likewise, in this study, six out of the eight individuals with TAMs were on tenofovir disoproxil fumarate-based regimen. Further, we observed that CD4 cell count was significantly lower (122 ± 72, mean ± SD) in individuals with TAM compared to non-TAM individuals (356 ± 387, mean ± SD). This is also consistent with reports from sub-Saharan African settings where individuals with previous exposure and drug resistance to thymidine analogues were at greater risk of clinical complication due to lower CD4 counts (Gregson et al., 2017). No correlation was detected between ART duration and frequency of DR mutations in this study. Although it is possible that even PI-naïve or INSTI-naïve patients can possess PI or INSTI resistance-associated mutations as a transmitted drug resistance, we did not detect any such mutations in the current study.

The preferred first-line regimen in Ghana during the study period was Tenofovir disoproxil fumarate + Lamivudine + Efavirenz [Ghana Health Service (GHS), National AIDS/STI Control Programme, 2016]. Thus, majority (56%) of ART-experienced participants were on Tenofovir + Lamivudine + Efavirenz regimen (Table 1). Other NRTIs used were zidovudine and lamivudine. While Efavirenz and Nevirapine were the NNRTI used by participants. Prevalence of resistance to Nevirapine (75%) and Efavirenz (71%) were the highest detected in this study. Further, 50% (14/28) of patient samples analyzed showed high−level resistance to multiple drugs, including Emtricitabine, lamivudine, Efavirenz, and Nevirapine. The current WHO guidelines for antiretroviral therapy recommend a first-line regimen consisting of dolutegravir with two NRTIs for all PLWH including adolescents, infants, and children (World Health Organization, 2021a). Despite the high level of M184V/I in this study, all sequences were susceptible to dolutegravir (Figure 2A); an indication that switching to dolutegravir-based regimens in Ghana would be beneficial. Dolutegravir-based regimens have been reported to be effective at maintaining viral suppression in treatment experienced PLWH carrying M184V/I (Ndashimye and Arts, 2021). Further, we show that 16 out of 28 (57%), ART-experienced participants had Genotypic Susceptibility Score (GSS) ≤1 and were highly resistant to at least two drugs prescribed for their treatment (Figure 2B). Prevalence of pretreatment/transmitted drug resistance in Ghana is relatively low (Nii-Trebi et al., 2013; Martin-Odoom et al., 2018; Deletsu et al., 2020; Obeng et al., 2020). However, in this study, two out of six sequences from ART naïve participants possessed DR mutations that conferred multiclass resistance. This indicates an increasing rate of pretreatment/transmitted drug resistance and raises concern. One ART naïve participant showed high-level resistance to all common NNRTI drugs. However, there were no TAMs detected in these two ART naïve sequences with DR mutations.

Our study is limited by our inability to ascertain the absence of previous ART usage in the patients we classified as ART naïve. Since undisclosed antiretroviral use in individuals presenting as treatment naive is common in Africa (Fogel et al., 2013), we may have classified some of these patients wrongly. On the other hand, the mutations observed in these ART-naïve patients could actually be due to the transmission of resistant strains within the population. Thus, calling for active surveillance of resistant strains among patients at the point of treatment initiation. Also, information on treatment interruptions/adherence was unavailable for participants, so we are unable to assess the impact of these on the resistance profiles observed.

Nonetheless, this study reports high-level resistance to NNRTI-based first-line regimens in Ghana in 2017 and provides evidence of the effectiveness of PI and INSTI in Ghana since no major DR mutations were detected against these drugs. The WHO currently recommends using dolutegravir in combination with an NRTI backbone as first-line regimen as well as second-line regimen for PLWH failing non-dolutegravir based regimen (World Health Organization, 2021a). This is yet to be extensively implemented in Ghana. Our data show that implementing this recommendation would most probably be the game changer in Ghana. Also, it is important to overcome the logistics challenges and implement routine viral load monitoring nationwide.

## Data availability statement

The data presented in the study are deposited in the GenBank repository (https://www.ncbi.nlm.nih.gov/genbank/), accession number ON863360-ON863428.

## Ethics statement

Ethical approval for this study was obtained from the Institutional Review Board of Noguchi Memorial Institute for Medical Research (NMIMR)-Ghana (approval number: 096/16-1; dated on May 3, 2017), and the Ethical Committee of National Institute of Infectious Diseases (NIID)-Japan (approval number: 685; dated on June 16, 2016). All study participants provided written informed consent to participate in this study.

## Author contributions

PP, EB, TK, MN, TMi, KI, WA, and TMa conceived and designed the experiments. GA-T, MA, SO, CA, DK, TA, and TMi contributed to demographic data and sample collection. PP, TA, CA, DK, and NN performed the experiments. PP, LR, and TK analyzed the data. PP, TK, and EB contributed to drafting the manuscript. HK, WA, and TMa critically reviewed and edited the manuscript and secured funding for this study. All authors contributed to the article and approved the submitted version.

## Funding

This study was supported by Japan Agency for Medical Research & Development (AMED) (grant numbers: JP22fk0410035, JP22fk0108125, JP22fk0108139, JP22jk0210002, JP22fk0410050, and JP21fk0410028), and AMED-JICA [the Science and Technology Research Partnership for Sustainable Development (SATREPS); JP20jm0110012].

## Conflict of interest

The authors declare that the research was conducted in the absence of any commercial or financial relationships that could be construed as a potential conflict of interest.

## Publisher’s note

All claims expressed in this article are solely those of the authors and do not necessarily represent those of their affiliated organizations, or those of the publisher, the editors and the reviewers. Any product that may be evaluated in this article, or claim that may be made by its manufacturer, is not guaranteed or endorsed by the publisher.

## References

[ref1] Aghokeng A. F. Kouanfack C. Laurent C. Ebong E. Atem-Tambe A. Butel C.. (2011). Scale-up of antiretroviral treatment in sub-Saharan Africa is accompanied by increasing HIV-1 drug resistance mutations in drug-naive patients. AIDS 25, 2183–2188. doi: 10.1097/QAD.0b013e32834bbbe9, PMID: 21860346

[ref2] ANRS (2015). ANRS AC11 resistance study group PCR and sequencing procedures: HIV-1. Version January 2015. Available at: https://hivfrenchresistance.org/wp-content/uploads/2021/10/ANRS-procedures.pdf (Accessed July 5, 2022).

[ref3] ANRS (2021). HIV-1 Genotypic Drug Resistance Interpretation Algorithm. Available at: https://hivfrenchresistance.org/hiv-french-resitance-tables-of-rules (Accessed February 1, 2022).

[ref4] Bennett D. E. Camacho R. J. Otelea D. Kuritzkes D. R. Fleury H. Kiuchi M.. (2009). Drug resistance mutations for surveillance of transmitted HIV-1 drug-resistance: 2009 update. PLoS One 4:e4724. doi: 10.1371/journal.pone.0004724, PMID: 19266092 PMC2648874

[ref5] Deletsu S. D. Maina E. K. Quaye O. Ampofo W. K. Awandare G. A. Bonney E. Y. (2020). High resistance to reverse transcriptase inhibitors among persons infected with human immunodeficiency virus type 1 subtype circulating recombinant form 02_AG in Ghana and on antiretroviral therapy. Medicine 99:e18777. doi: 10.1097/MD.0000000000018777, PMID: 32049783 PMC7035011

[ref6] Delgado E. Ampofo W. K. Sierra M. Torpey K. Pérez-Álvarez L. Bonney E. Y.. (2008). High prevalence of unique recombinant forms of HIV-1 in Ghana: molecular epidemiology from an antiretroviral resistance study. JAIDS J. Acquir. Immune Defic. Syndr. 48, 599–606. doi: 10.1097/QAI.0b013e3181806c0e, PMID: 18645511

[ref7] Fogel J. M. Wang L. Parsons T. L. Ou S.-S. Piwowar-Manning E. Chen Y.. (2013). Undisclosed antiretroviral drug use in a multinational clinical trial (HIV prevention trials network 052). J. Infect. Dis. 208, 1624–1628. doi: 10.1093/infdis/jit390, PMID: 23908493 PMC3805242

[ref8] Ghana Health Service (GHS), National AIDS/STI Control Programme (2016). Guidelines for Antiretroviral Therapy in Ghana. 6th Edn.

[ref9] Gregson J. Kaleebu P. Marconi V. C. van Vuuren C. Ndembi N. Hamers R. L.. (2017). Occult HIV-1 drug resistance to thymidine analogues following failure of first-line tenofovir combined with a cytosine analogue and nevirapine or efavirenz in sub Saharan Africa: a retrospective multi-centre cohort study. Lancet Infect. Dis. 17, 296–304. doi: 10.1016/S1473-3099(16)30469-8, PMID: 27914856 PMC5421555

[ref10] Gupta R. K. Gregson J. Parkin N. Haile-Selassie H. Tanuri A. Andrade Forero L.. (2018). HIV-1 drug resistance before initiation or re-initiation of first-line antiretroviral therapy in low-income and middle-income countries: a systematic review and meta-regression analysis. Lancet Infect. Dis. 18, 346–355. doi: 10.1016/S1473-3099(17)30702-8, PMID: 29198909 PMC5835664

[ref11] Hamers R. L. Sigaloff K. C. E. Wensing A. M. Wallis C. L. Kityo C. Siwale M.. (2012). Patterns of HIV-1 drug resistance After first-line antiretroviral therapy (ART) failure in 6 sub-Saharan African countries: implications for second-line ART strategies. Clin. Infect. Dis. 54, 1660–1669. doi: 10.1093/cid/cis254, PMID: 22474222

[ref12] Hemelaar J. Elangovan R. Yun J. Dickson-Tetteh L. Fleminger I. Kirtley S.. (2019). Global and regional molecular epidemiology of HIV-1, 1990–2015: a systematic review, global survey, and trend analysis. Lancet Infect. Dis. 19, 143–155. doi: 10.1016/S1473-3099(18)30647-9, PMID: 30509777

[ref13] Henerico S. Mikasi S. G. Kalluvya S. E. Brauner J. M. Abdul S. Lyimo E.. (2022). Prevalence and patterns of HIV drug resistance in patients with suspected virological failure in North-Western Tanzania. J. Antimicrob. Chemother. 77, 483–491. doi: 10.1093/jac/dkab406, PMID: 35107140 PMC8809186

[ref14] Kotaki T. Khairunisa S. Q. Sukartiningrum S. D. Witaningrum A. M. Rusli M. Diansyah M. N.. (2014). Detection of drug resistance-associated mutations in human immunodeficiency virus type 1 integrase derived from drug-naive individuals in Surabaya, Indonesia. AIDS Res. Hum. Retrovir. 30, 489–492. doi: 10.1089/AID.2013.0271, PMID: 24328535

[ref15] Liu T. F. Shafer R. W. (2006). Web resources for HIV type 1 genotypic-resistance test interpretation. Clin. Infect. Dis. 42, 1608–1618. doi: 10.1086/503914, PMID: 16652319 PMC2547473

[ref16] Martin-Odoom A. Brown C. A. Odoom J. K. Bonney E. Y. Ntim N. A. A. Delgado E.. (2018). Emergence of HIV-1 drug resistance mutations in mothers on treatment with a history of prophylaxis in Ghana. Virol. J. 15, 143. doi: 10.1186/s12985-018-1051-2, PMID: 30223845 PMC6142311

[ref17] Melikian G. L. Rhee S.-Y. Taylor J. Fessel W. J. Kaufman D. Towner W.. (2012). Standardized comparison of the relative impacts of HIV-1 reverse transcriptase (RT) mutations on nucleoside RT inhibitor susceptibility. Antimicrob. Agents Chemother. 56, 2305–2313. doi: 10.1128/AAC.05487-11, PMID: 22330916 PMC3346663

[ref18] Melikian G. L. Rhee S.-Y. Varghese V. Porter D. White K. Taylor J.. (2014). Non-nucleoside reverse transcriptase inhibitor (NNRTI) cross-resistance: implications for preclinical evaluation of novel NNRTIs and clinical genotypic resistance testing. J. Antimicrob. Chemother. 69, 12–20. doi: 10.1093/jac/dkt316, PMID: 23934770 PMC3861329

[ref001] Ndashimye E. Arts E. J. (2021). Dolutegravir response in antiretroviral therapy naïve and experienced patients with M184V/I: impact in low-and middle-income settings. Int. J. Infect. Dis. 105, 298–303. doi: 10.1016/j.ijid.2021.03.018, PMID: 33722682

[ref19] Nii-Trebi N. I. Brandful J. A. M. Ibe S. Sugiura W. Barnor J. S. Bampoh P. O.. (2017). Dynamic HIV-1 genetic recombination and genotypic drug resistance among treatment-experienced adults in northern Ghana. J. Med. Microbiol. 66, 1663–1672. doi: 10.1099/jmm.0.000621, PMID: 29068286

[ref20] Nii-Trebi N. I. Ibe S. Barnor J. S. Ishikawa K. Brandful J. A. M. Ofori S. B.. (2013). HIV-1 drug-resistance surveillance among treatment-experienced and -naïve patients after the implementation of antiretroviral therapy in Ghana. PLoS One 8:e71972. doi: 10.1371/journal.pone.0071972, PMID: 23977189 PMC3747072

[ref21] Obeng B. M. Bonney E. Y. Asamoah-Akuoko L. Nii-Trebi N. I. Mawuli G. Abana C. Z.-Y.. (2020). Transmitted drug resistance mutations and subtype diversity amongst HIV-1 sero-positive voluntary blood donors in Accra, Ghana. Virol. J. 17, 114. doi: 10.1186/s12985-020-01386-y, PMID: 32709248 PMC7378406

[ref22] Ode H. Matsuda M. Matsuoka K. Hachiya A. Hattori J. Kito Y.. (2015). Analyses of the HIV-1 near-full-length genome With Illumina MiSeq. Front. Microbiol. 6:1258. doi: 10.3389/fmicb.2015.01258, PMID: 26617593 PMC4641896

[ref23] Pineda-Peña A.-C. Faria N. R. Imbrechts S. Libin P. Abecasis A. B. Deforche K.. (2013). Automated subtyping of HIV-1 genetic sequences for clinical and surveillance purposes: performance evaluation of the new REGA version 3 and seven other tools. Infect. Genet. Evol. 19, 337–348. doi: 10.1016/j.meegid.2013.04.032, PMID: 23660484

[ref24] Quercia R. Perno C.-F. Koteff J. Moore K. McCoig C. Clair M. S. T.. (2018). Twenty-five years of lamivudine: current and future use for the treatment of HIV-1 infection. J. Acquir. Immune Defic. Syndr. 78, 125–135. doi: 10.1097/QAI.0000000000001660, PMID: 29474268 PMC5959256

[ref25] Siepel A. C. Halpern A. L. Macken C. Korber B. T. M. (1995). A computer program designed to screen rapidly for HIV type 1 Intersubtype recombinant sequences. AIDS Res. Hum. Retrovir. 11, 1413–1416. doi: 10.1089/aid.1995.11.1413, PMID: 8573400

[ref26] Tzou P. L. Rhee S.-Y. Descamps D. Clutter D. S. Hare B. Mor O.. (2020). Integrase strand transfer inhibitor (INSTI)-resistance mutations for the surveillance of transmitted HIV-1 drug resistance. J. Antimicrob. Chemother. 75, 170–182. doi: 10.1093/jac/dkz417, PMID: 31617907 PMC7850029

[ref27] UNAIDS (2014). Fast-Track—Ending the AIDS Epidemic by 2030. Joint United Nations Programme on HIV/AIDS Available at: https://www.unaids.org/sites/default/files/media_asset/JC2686_WAD2014report_en.pdf (Accessed April 21, 2022).

[ref28] UNAIDS (2019). Country progress report—Ghana. Available at: https://www.unaids.org/sites/default/files/country/documents/GHA_2019_countryreport.pdf (Accessed April 22, 2022).

[ref29] Villa G. Phillips R. O. Smith C. Stockdale A. J. Ruggiero A. Beloukas A.. (2018). Drug resistance outcomes of long-term ART with tenofovir disoproxil fumarate in the absence of virological monitoring. J. Antimicrob. Chemother. 73, 3148–3157. doi: 10.1093/jac/dky281, PMID: 30032305 PMC6198639

[ref30] Wensing A. M. Calvez V. Ceccherini-Silberstein F. Charpentier C. Günthard H. F. Paredes R.. (2019). 2019 update of the drug resistance mutations in HIV-1. Top. Antivir. Med. 27, 111–121. PMID: 31634862 PMC6892618

[ref31] Whitcomb J. M. Parkin N. T. Chappey C. Hellmann N. S. Petropoulos C. J. (2003). Broad nucleoside reverse-transcriptase inhibitor cross-resistance in human immunodeficiency virus type 1 clinical isolates. J. Infect. Dis. 188, 992–1000. doi: 10.1086/378281, PMID: 14513419

[ref32] Woods C. K. Brumme C. J. Liu T. F. Chui C. K. S. Chu A. L. Wynhoven B.. (2012). Automating HIV drug resistance genotyping with RECall, a freely accessible sequence analysis tool. J. Clin. Microbiol. 50, 1936–1942. doi: 10.1128/JCM.06689-11, PMID: 22403431 PMC3372133

[ref33] World Health Organization (2019). HIV DRUG RESISTANCE REPORT 2019. Geneva, Switzerland Available at: https://apps.who.int/iris/rest/bitstreams/1238263/retrieve (Accessed April 21, 2022).

[ref34] World Health Organization (2021a). Consolidated guidelines on HIV prevention, testing, treatment, service delivery and monitoring: recommendations for a public health approach. 2021 update. Geneva: World Health Organization Available at: https://apps.who.int/iris/handle/10665/342899 (Accessed April 25, 2022). 34370423

[ref35] World Health Organization (2021b). HIV/AIDS—Fact Sheet. HIVAIDS - Fact Sheet. Available at: https://www.who.int/news-room/fact-sheets/detail/hiv-aids (Accessed April 22, 2022).

