# Supplementary material for: High-level resistance to non-nucleos(t)ide reverse transcriptase inhibitor based first-line antiretroviral therapy in Ghana; A 2017 study
Source: Front Microbiol. 2022 Aug 25;13:973771. doi: 10.3389/fmicb.2022.973771 (PMC9459847; doi:10.3389/fmicb.2022.973771)
Supplement: Supplementary file 1 [file Table_1.DOCX]

**Supplementary Table 1. The primers used for the reverse transcription PCR and the nested PCR.**

| **Name** | **Position** | **Target** | **PCR** | **Primer sequence(5'>3')** |
| --- | --- | --- | --- | --- |
| 5PROT1 | 2082-2109 | PR | Round 1 | TAATTTTTTAGGGAAGATCTGGCCTTCC |
| 3PROT1 | 2703-2734 | PR | Round 1 | GCAAATACTGGAGTATTGTATGGATTTTCAGG |
| 5PROT2 | 2136-2163 | PR | Round 2 | TCAGAGCAGACCAGAGCCAACAGCCCCA |
| 3PROT2 | 2621-2650 | PR | Round 2 | AATGCTTTTATTTTTTCTTCTGTCAATGGC |
| MJ3 | 2480-249 | RT | Round 1 | AGTAGGACCTACACCTGTCA |
| MJ4 | 3399-3420 | RT | Round 1 | CTGTTAGTGCTTTGGTTCCTCT |
| A35 | 2530-2564 | RT | Round 2 | TTGGTTGCACTTTAAATTTTCCCATTAGTCCTATT |
| NE135 | 3299-3334 | RT | Round 2 | CCTACTAACTTCTGTATGTCATTGACAGTCCAGCT |
| DRIN01 | 4039-4060 | IN | Round 1 | CAGACTCACAATATGCATTAGG |
| DRIN02 | 5243-5264 | IN | Round 1 | CCTGTATGCAGACCCCAATATG |
| DRIN05 | 4146-4168 | IN | Round 2 | CTGGCATGGGTACCAGCACACAA |
| DRIN04 | 5195-5217 | IN | Round 2 | TAGTGGGATGTGTACTTCTGAAC |

**Supplementary Figure 1**. Flow chart summarizing the number of successful sequences from respective ART groups; protease (PR), reverse transcriptase (RT), and integrase (IN)

Links to databases and software tools used for analyzing data in the study:

Sequence editing and assembly; RECall

<https://recall.bccfe.ca/wiki>

Subtyping ; REGA and RIP

<https://www.genomedetective.com/app/typingtool/hiv/>

<https://www.hiv.lanl.gov/content/sequence/RIP/RIP.html>

Interpretation of drug resistance mutations; Stanford HIV database

<https://hivdb.stanford.edu/>

Phylogenetic inference of HIV-1 subtypes; MEGA11 and Los Alamos database

<https://www.hiv.lanl.gov/content/sequence/NEWALIGN/align.html>

<https://www.megasoftware.net/>
